# Department of Public Health

**Published:** 1898-02

**Authors:** 


### Abstracts and Selections.

### Department of Public Health.

The following editorial (of November 6th, 1897) from the *Journal of the American Medical Association* on a Department of Public Health, explains the object and purpose of a bill for a Department of Public Health, which is endorsed by the American Medical Association and the American Public Health Association and which is to be presented to Congress at its coming session:

"It is with pleasure that we inform the members of the Association that the Association Bill to establish a Department of Public Health, and define its duties, has bean approved in its entirety by the American Public Health Association at its last meeting at Philadelphia, and that body will co-operate with the Association and endeavor to have it placed upon the statute books.

"There is some misapprenhension in regard to the bill, as appears by comments in certain newspapers, which should be relieved. In the first place, the Association yielded to the inevitable, no longer insisting that the head of the new department should be a member of the cabinet, to which position it heretofore found there were insuperable objections; objections which would indeed prevent the passage of the bill should it be presented. The committee, therefore, have wisely framed their bill on the lines of the Department of Agriculture, and there is little question but that in time the objections which now seem so unsurmountable will fade before the pressure of public opinion. However, the present proposition establishes the foundation and paves the way, but whether the period of trial will be long or short is of comparatively little consequence to the establishment of the new department itself, which can run just as well if its head does not have that full measure of civic honor that the friends of the department would like to see him have. At present, so far as the public is concerned, it is the efficiency of the service, and the thoroughness with which the new service shall cover the sanitary field, that they look to.

"Some misapprehension has been felt and indeed expressed in some of the daily newspapers, to the effect that this measure was simply a revival of the old National Board of Public Health; that it was the establishing of a national quarantine with greater powers; and to such we must reply that these misapprehensions are not founded on fact. In the first place, this bill for the first time provides for thorough correspondence or conference to secure the co-operation of State, municipal and local Boards of Health in establishing and maintaining an efficient and accurate system of notification of the existence and progress of contagious and infectious diseases and of vital statistics in the United States. While the proposed bill goes further than any existing law in regard to the investigation of the cause of disease and the best means of its prevention, it makes no new regulation regarding quarantine. It takes the quarantine as it finds it and simply transfers from the secretary of the treasury, a purely financial and commercial officer, the duties and supervision of so much of the work of the Marine Hospital Service as is included in this law, and so far as the service is concerned—an organization which, we are informed, attempted vainly to stem the tide of public opinion at Philadelphia by opposing the adoption of the measure—we have to say that it will make no difference to the officers of the service, so far as their duties are concerned, except that the head of the service, instead of reporting to the secretary of the treasury, will report to the Commissioner of Public Health, that is to say, that he will in future, if this bill establishing a Department of Public Health shall become a law, report to some one presumably competent to give him advice and instruction—a person sadly needed when we consider the mismanagement of the present epidemic. The existing quarantine laws then are transferred bodily from the Treasury Department to the Department of Public Health, a transfer which every dictate of humanity and of good government seems to suggest. The bill goes further than any existing law in its utilization of the machinery of the present departments of government, and the great skill and adroitness which the committee of the Association have prepared it, leaves all the departments and bureaus of the government in the same departments where they are now found. Is simply provides for the better utilization of the information at their disposal, and affords a place for its collation and dissemination. The bill also codifies all existing laws. That it is perfect in all details, we suppose is not to be expected so long as human nature itself is not perfect, but it is as near perfection as possible without further trial to secure it. We shall not only sincerely hope that every member of the Association will use his utmost influence to secure the passage of this wise, salutary and beneficent law, but that he will immediately write to his member of Congress, and his senators, giving them the facts in regard to it, pointing out that the bill, as already stated, differs from the old National Board of Health Act, inasmuch as it provides for a representative of every State Board of Health in the Union; that it consolidates the existing United States statutes; that it maintains all good and effective laws now in force, including the National Quarantine Act, and the Interstate Quarantine Act, and that it provides for the utilization of information in the hands of the various departments, and affords a proper and suitable place for dissemination and promulgation. The bill unloads the overloaded Treasury Department from duties which, under the existing state of affairs, were only nominal, and places them where care and supervision can be exercised. As the Marine Hospital service is not interfered with, as to the officer to whom the chief of the bureau shall report, it is seen that the opposition to the measure is not only gratuitous and unnecessary, but unwise and unscientific. We, however, are of the opinion that this alleged opposition is confined to a limited number of officers who are compelled to act under the instruction of the present chief, who is said to view any change of whatever character in the existing regime as a bad one."

